# Low resolution scans can provide a sufficiently accurate, cost- and time-effective alternative to high resolution scans for 3D shape analyses

**DOI:** 10.7717/peerj.5032

**Published:** 2018-06-22

**Authors:** Ariel E. Marcy, Carmelo Fruciano, Matthew J. Phillips, Karine Mardon, Vera Weisbecker

**Affiliations:** 1 School of Biological Sciences, University of Queensland, Brisbane, Queensland, Australia; 2 Institut de biologie de l’Ecole normale supérieure, Ecole normale supérieure, Université Paris, Paris, France; 3 School of Earth, Environmental and Biological Sciences, Queensland University of Technology, Brisbane, Queensland, Australia; 4 Centre for Advanced Imaging, University of Queensland, Brisbane, Queensland, Australia; 5 National Imaging Facility, University of Queensland, Brisbane, Queensland, Australia

**Keywords:** Geometric morphometrics, Shape variation, Photogrammetry, *Pseudomys delicatulus*, Geomorph, Systematic error, Random error, Generalized procrustes analysis

## Abstract

**Background:**

Advances in 3D shape capture technology have made powerful shape analyses, such as geometric morphometrics, more feasible. While the highly accurate micro-computed tomography (µCT) scanners have been the “gold standard,” recent improvements in 3D surface scanners may make this technology a faster, portable, and cost-effective alternative. Several studies have already compared the two devices but all use relatively large specimens such as human crania. Here we perform shape analyses on Australia’s smallest rodent to test whether a 3D scanner produces similar results to a µCT scanner.

**Methods:**

We captured 19 delicate mouse (*Pseudomys delicatulus*) crania with a µCT scanner and a 3D scanner for geometric morphometrics. We ran multiple Procrustes ANOVAs to test how variation due to scan device compared to other sources such as biologically relevant variation and operator error. We quantified operator error as levels of variation and repeatability. Further, we tested if the two devices performed differently at classifying individuals based on sexual dimorphism. Finally, we inspected scatterplots of principal component analysis (PCA) scores for non-random patterns.

**Results:**

In all Procrustes ANOVAs, regardless of factors included, differences between individuals contributed the most to total variation. The PCA plots reflect this in how the individuals are dispersed. Including only the symmetric component of shape increased the biological signal relative to variation due to device and due to error. 3D scans showed a higher level of operator error as evidenced by a greater spread of their replicates on the PCA, a higher level of multivariate variation, and a lower repeatability score. However, the 3D scan and µCT scan datasets performed identically in classifying individuals based on intra-specific patterns of sexual dimorphism.

**Discussion:**

Compared to µCT scans, we find that even low resolution 3D scans of very small specimens are sufficiently accurate to classify intra-specific differences. We also make three recommendations for best use of low resolution data. First, we recommend that extreme caution should be taken when analyzing the asymmetric component of shape variation. Second, using 3D scans generates more random error due to increased landmarking difficulty, therefore users should be conservative in landmark choice and avoid multiple operators. Third, using 3D scans introduces a source of systematic error relative to µCT scans, therefore we recommend not combining them when possible, especially in studies expecting little biological variation. Our findings support increased use of low resolution 3D scans for most morphological studies; they are likely also applicable to low resolution scans of large specimens made in a medical CT scanner. As most vertebrates are relatively small, we anticipate our results will bolster more researchers in designing affordable large scale studies on small specimens with 3D surface scanners.

## Introduction

**Figure 1:**
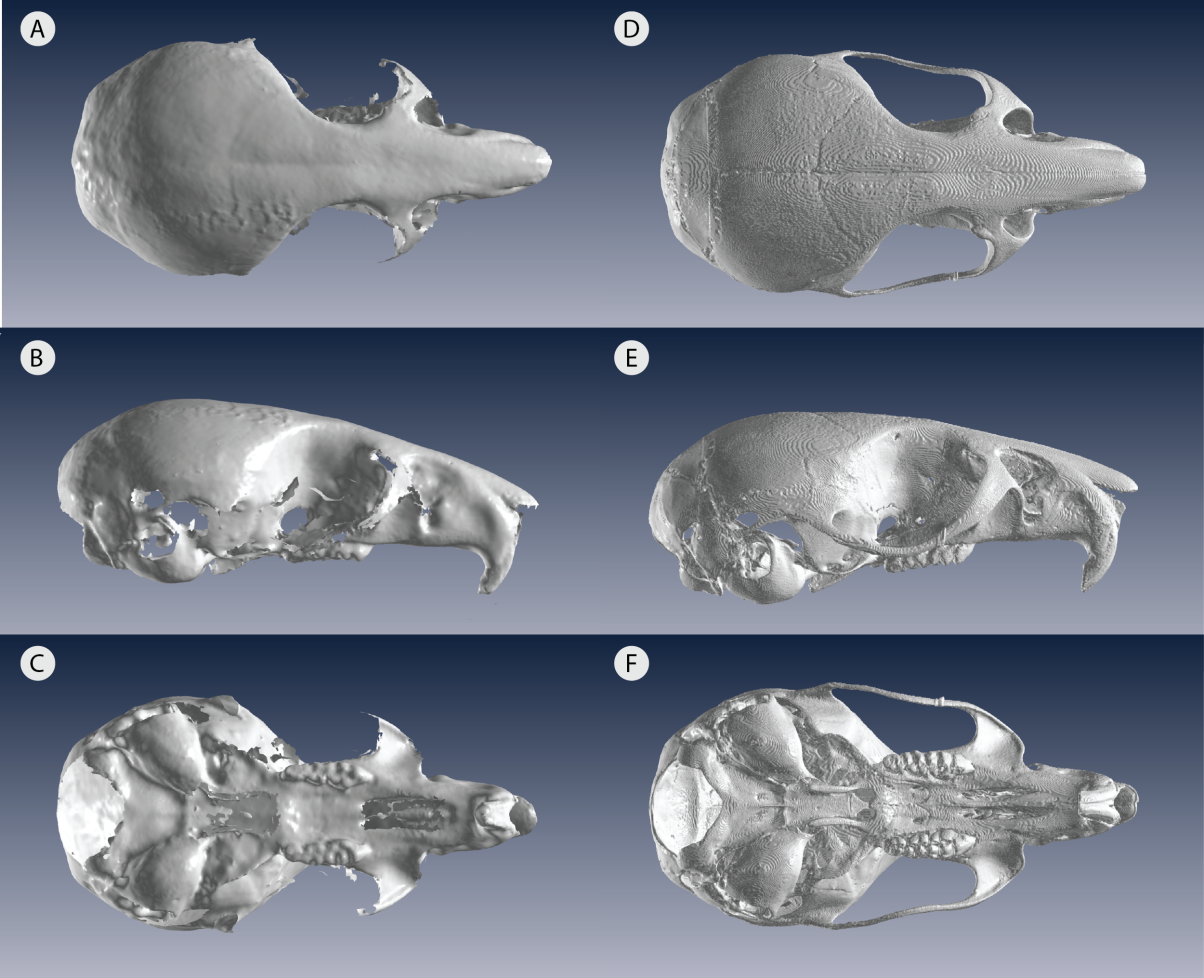
Low resolution 3D surface scans compared to µCT scans of the same delicate mouse crania. 3D scans of (A) dorsal view (B) lateral view and (C) ventral view compared to µCT scans of (D) dorsal view (E) lateral view and (F) ventral view. All crania are rendered in Viewbox v. 4.0.

An organism’s shape reveals many facets of its biology, including its evolution, ecology, and functional morphology. In the past three decades, geometric morphometrics has revolutionized the field of shape research with better analysis and visualization of shape complexity (Rohlf & Marcus, 1993; Zelditch, Swiderski & Sheets, 2012). As imaging technology continues to advance, three-dimensional (3D) data have become extremely common in geometric morphometric studies, especially in the cases in which 2D data poorly represent the actual 3D object (Buser, Sidlauskas & Summers, 2017; Cardini, 2014; Fruciano, 2016; Reig, 1996). 3D capture methods include very high resolution yet high cost micro-computed tomography (µCT) scanners, which usually require time-intensive sectioning with specialized software. In contrast, 3D surface scanners offer lower acquisition costs as well as faster scanning and processing, but has the disadvantage of generally lower resolution, which limits its use on very small specimens (Fig. 1). For confident use of surface scans in small specimens, it is therefore important to assess the measurement error introduced by choosing a 3D surface scanner for geometric morphometrics.

Most vertebrates would be considered small, for example about two thirds of mammals are below 10 kg (Weisbecker & Goswami, 2010), which would translate to small skeletal specimens. Therefore, morphometric studies proposing large sample sizes must be very well funded to use a µCT scanner or have a low-cost option, such as a 3D surface scanner. Previous studies have compared µCT scans to 3D surface scans, however, these were all done in large animals, primarily primates (Badawi-Fayad & Cabanis, 2007; Fourie et al., 2011; Katz & Friess, 2014; Robinson & Terhune, 2017; Sholts et al., 2010; Slizewski, Friess & Semal, 2010). While these studies found low error and high repeatability in 3D surface scans similar to µCT scans, there was a suggestion that higher error occurred in the sample’s smaller specimens (Badawi-Fayad & Cabanis, 2007; Fourie et al., 2011). Other recent studies have conducted 3D geometric morphometric studies on small vertebrate skulls but nearly all have relied exclusively on µCT scanning (Cornette et al., 2013; Evin, Horacek & Hulva, 2011). The only exception we are aware of is Muñoz Muñoz, Quinto-Sánchez & González-José (2016), which successfully used photogrammetry—a technique combining 2D photographs into a 3D model—to analyze domestic mouse skulls, *Mus musculus domesticus* (Linnaeus, 1758). Photogrammetry, like 3D surface scanning, is a low-cost alternative to µCT and comes with its own trade-offs in time and scan resolution (Katz & Friess, 2014). Compared to the new generation of blue light surface scanners, photogrammetry requires more time for image acquisition and for file processing (Katz & Friess, 2014). A previous study on a single macaque specimen reported inconsistent levels of error across operators and scanners, which contributed to the lack of general pattern for differences across scanners/resolutions (Shearer et al., 2017). However, using an interspecific dataset, (Fruciano et al., 2017) reported higher repeatability for the higher resolution scans and 2.07–11.26% of total variance due to scan type (depending on device, operator and landmark set combination). We expect that small specimens would exacerbate any variation due to device and the interaction of device with other factors, such as landmark choice and operator. More work comparing these different methods—µCT scanning, 3D surface scanning, and photogrammetry—will allow researchers to make an informed decision. For example, for those with time constraints in museum collections, a fast 3D surface scanner may be the best option if the resolution is suitable for specimen size.

The lower resolution of 3D surface scanners may increase both random and systematic measurement error, which is exacerbated by small specimens because operators may have more difficulty identifying landmark locations (Arnqvist & Martensson, 1998; Fruciano, 2016). Random error increases variance without changing the mean; this “noise” dilutes biologically informative patterns and, in principle, decreases statistical power (Arnqvist & Martensson, 1998; Fruciano, 2016). By contrast, systematic error is non-randomly distributed, thus changing the mean and introducing bias to the data (Arnqvist & Martensson, 1998; Fruciano, 2016). Error assessment can be done with repeated measures of the same individuals (e.g., Fruciano et al., 2017; Muñoz Muñoz & Perpiñán, 2010; Robinson & Terhune, 2017) or by comparison to a “gold standard” or ideal representation of the specimens (Fruciano, 2016; Slizewski, Friess & Semal, 2010; Williams & Richtsmeier, 2003). Repeated measure designs can uncover this systematic error, for example, if one 3D capture method differs from another in a specific, non-random, pattern (Fruciano, 2016; Fruciano et al., 2017). Furthermore, designs including repeated measures of the same individuals allow partitioning of variance into components, quantifying error due to scan device as compared to biologically-relevant sources of variation such as asymmetry (Fruciano, 2016; Klingenberg, Barluenga & Meyer, 2002; Klingenberg & McIntyre, 1998).

**Figure 2:**
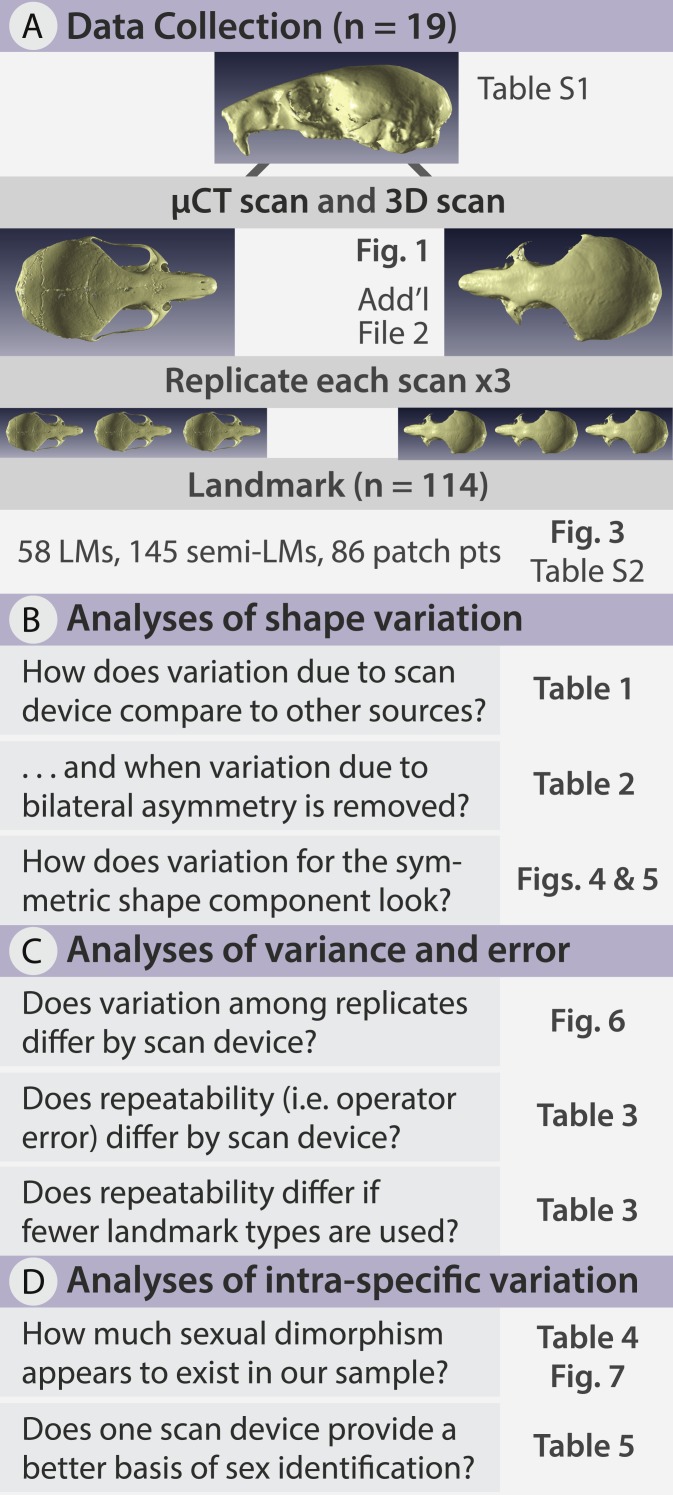
Methods flow diagram highlighting the relationship between our questions and our analyses. (A) All delicate mouse (*Pseudomys delicatulus*) crania were sourced from the Queensland Museum in Brisbane, Australia. Landmarks (LMs) capture homologous points, semi-landmarks (semi-LMs) capture curves between landmarks, and patch points capture surfaces between landmarks and semi-landmarks. (B–D) These sections of questions and associated figure and table numbers summarize how we organize the paper, particularly the Results, into three sets of related analyses.

In this study, we quantify the error introduced by studying specimens of a size at the very lower limits of commonly used portable surface scanners’ resolution. This situation could also arise when using relatively large specimens, which are nonetheless at the lower limit of a medical CT scanner’s resolution for example. We test whether the complex shape of very small specimens can be adequately captured using an HDI109 3D surface scanner (LMI Technologies Inc., Vancouver, Canada) with a stated resolution of 80 µm as compared to a µCT scanner with a resolution of 28 µm. To do so, we use the delicate mouse, *Pseudomys delicatulus* (Gould, 1842), one of the smallest rodents in the world with a 55–75 mm head-and-body length (Breed & Ford, 2007). The miniscule *P. delicatulus* crania (∼20 mm) are at the edge of the HDI109 3D surface scanner’s range thus providing an extreme test of this scanning device (Figs. 1 and 2). First, we tested whether variation due to scanning device compared to other sources of variation (Fig. 2B). We also asked whether removing asymmetric variation, a common practice in morphological studies when asymmetry is not of interest, changed the results. Second, we tested whether the scanning devices differed in shape variance and in operator error (as measured by repeatability) (Fig. 2C). We also explored how including different types of landmarks impacted repeatability. Finally, we tested whether the shape variation due to scanning device was large enough to impact a small study of intra-specific shape variation using the biologically relevant signal of sexual dimorphism (Fig. 2D).

## Methods

### Data collection

We selected 19 adult individuals, male and female, of *Pseudomys delicatulus* from the Queensland Museum in Brisbane, Australia (specimen numbers and sexes in Table S1). The cranium from each individual was scanned at the Centre for Advanced Imaging at the University of Queensland in a µCT scanner (Siemens Inveon PET/CT scanner, Munich, Germany). The scanner was operated at 80 kV energy, 250 µA intensity with 540 projections per 360°, a medium-high magnification with bin 2 was applied, and 2,000 ms exposure time. The samples were scanned at a nominal isotropic resolution of 28 µm. The data were reconstructed using a Feldkamp conebeam back-projection algorithm provided by an Inveon Acquisition workstation from Siemens (IAW version 2.1, Munich, Germany). Surface models were obtained using Mimics Research version 20.0.

Each cranium was also scanned by a HDI109 blue light surface scanner (LMI Technologies Inc., Vancouver, Canada) with a resolution of 80 µm. For brevity, we will refer to this method as 3D scanning. For this method, the cranium was placed on a rotary table providing the scanner with 360° views. To capture the entire shape, the cranium was scanned in three different orientations: one ventral view with the cranium resting on the frontals and two dorsal views with the cranium tipped to each side, resting on an incisor, auditory bulla, and zygomatic arch. To assist others in replicating our HDI109 3D surface scanning on small specimens, we have included a Standard Operating Procedure with our settings (Supplemental Information 1).

**Figure 3:**
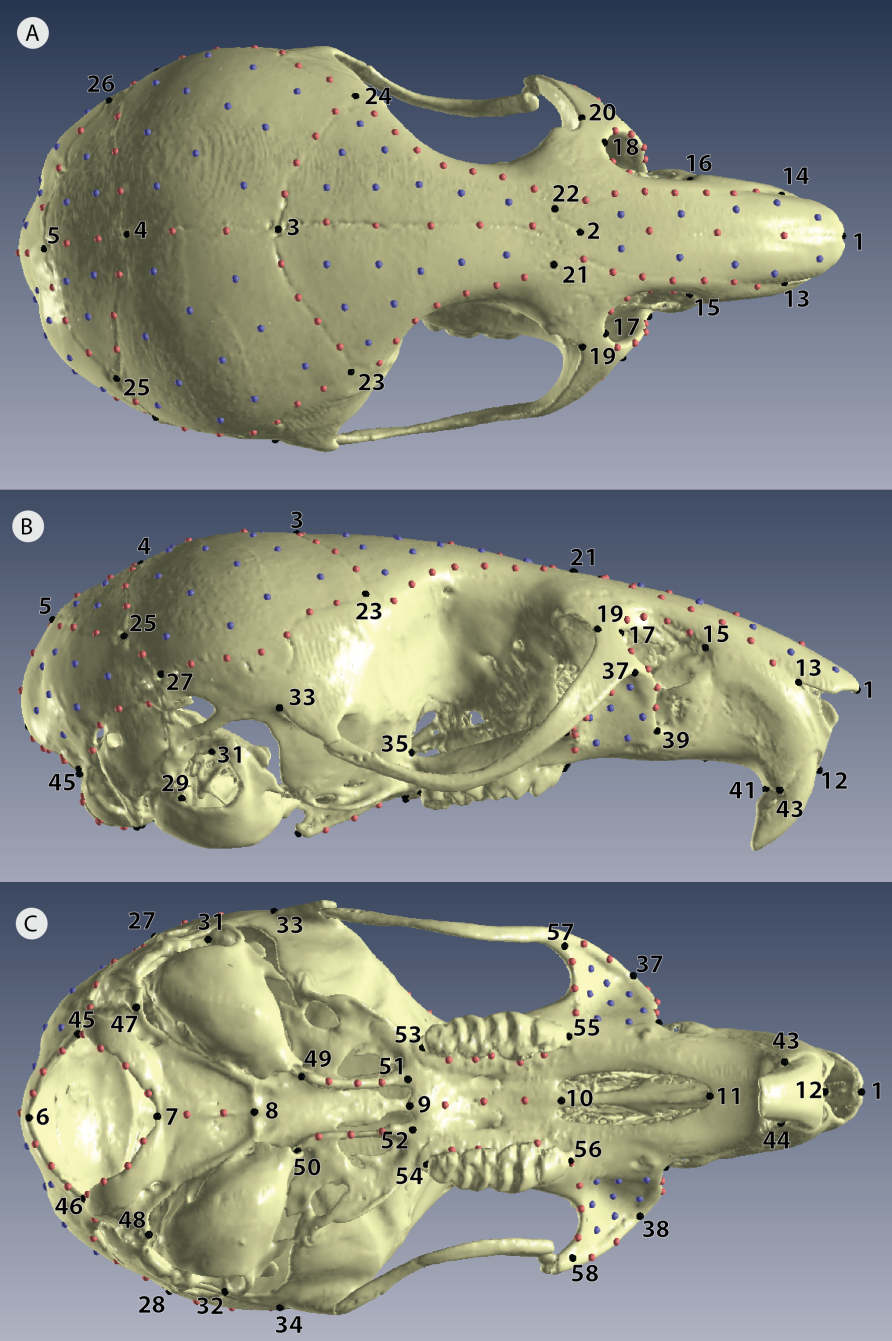
Positions of landmarks for geometric morphometric analyses. Locations of fixed landmarks (black points), sliding semi-landmarks (red points) and sliding surface patches (purple points) on a µCT scanned individual. (A) Dorsal view of the cranium. (B) Lateral view. (C) Ventral view. Definitions are given in Table S2.

After scanning every individual with both scan methods, we then replicated each 3D model three times so that each individual was represented by six replicates, giving a total sample of 114 3D models (Fig. 2A). Each 3D model was landmarked in Viewbox version 4.0 (dHAL software, Kifissia, Greece; http://www.dhal.com; Polychronis et al., 2013). To capture shape, we placed 58 fixed landmarks, 145 semi-landmarks on curves, and 86 patch points (points that during sliding are allowed to slide across a 3D surface defined by the 3D model and semi-landmark borders) for a total of 289 points (Fig. 3, Table S2). We used the template feature in Viewbox to semi-automate the placement of semi-landmarks on curves and to fully automate the placement of patch points. Our landmark design covered most important biological structures except for the zygomatic arch (Fig. 3); we avoided this fine structure because dehydration and loss of support from surrounding muscles during skeletonization almost certainly causes specimen preparation error (Schmidt et al., 2010; Yezerinac, Lougheed & Handford, 1992).

### Data analysis

The landmark coordinates for all 114 3D models were aligned using a generalized Procrustes analysis followed by projection to the tangent space, as implemented in the R package *geomorph* (v. 3.0.5) (Adams, Collyer & Sherratt, 2016; Adams & Otarola-Castillo, 2013). Generalized Procrustes analysis of each set of landmark coordinates removes differences in size, position, and orientation, leaving only shape variation (Rohlf & Slice, 1990). Semi-landmarks and patches were permitted to slide along their tangent directions to minimize Procrustes distance between 3D models (Gunz, Mitteroecker & Bookstein, 2005). The resulting Procrustes tangent coordinates were used as shape variables in all subsequent shape analyses. All our statistical analyses were performed either in R (v. 3.3.3) using the R packages *geomorph* (v. 3.0.5) (Adams 2016; (Adams & Otarola-Castillo, 2013) and *Morpho* (v. 2.5.1) (Schlager, 2017) or in MorphoJ (v. 1.06d) (Klingenberg, 2011).

First, asymmetry is a known source of variation within a sample (Klingenberg, Barluenga & Meyer, 2002), so we tested for it with MorphoJ’s Procrustes ANOVA function and subsequently removed it (Fig. 2B). Isolating the symmetric component of shape has been undertaken in other 3D surface scanner studies where operator and device error have been of the same magnitude as asymmetric error (Fruciano et al., 2017). Variation due to asymmetry is more impacted by operator error because of its smaller effect sizes compared to variation among individuals (Fruciano, 2016; Fruciano et al., 2017; Klingenberg et al., 2010; Leamy & Klingenberg, 2005). This suggests that low resolution studies on asymmetry would be negatively impacted. For this reason, we performed most subsequent analyses on the symmetric shape component, with a few exceptions performed for comparison. We then performed a PCA on the symmetric shape variables to visualize the variation between individuals, within scan method replicates, and between scan method replicates. As an exploratory analysis, PCA can help intuitively visualize both random error (greater spread of one scan method replicate compared to the other) and systematic error (repeated pattern of one scan method shifting relative to another). However, further analyses are necessary to quantify these sources of error.

Second, our replicate design allowed us to assess whether an operator digitizing scans from one device was more variable in landmark placement than when digitizing scans from the other device (Fig. 2C). We did so by computing the Procrustes variance for each individual/device combination. In *geomorph*, Procrustes variances are calculated for each set of observations (i.e., replicates) as the sum of the diagonal elements of the set’s covariance matrix divided by the number of observations (Adams, Collyer & Sherratt, 2016; Zelditch, Swiderski & Sheets, 2012). We computed Procrustes variance for each combination of individual and device so that Procrustes variance reflected only variation due to digitization. We then compared Procrustes variance between devices using a box plot and the permutational procedure implemented in *geomorph*. Next we quantified digitization consistency by computing repeatability for each device using the analogue of the intraclass correlation coefficient computed with the Procrustes ANOVA mean squares, as suggested by Fruciano (2016). This value is normally between 0 and 1, with values close to 1 indicating much larger variation due to the factor used in computing the Procrustes ANOVA (in our case, variation among individuals) compared to residual variation (in our case, variation among digitizations). In other words, comparing repeatability between devices gives similar information to that obtained by the box plots of Procrustes variance but on a more easily interpretable scale from 0 to 1. We repeated our computations of repeatability for subsets of the data to test whether introducing semi-landmarks on curves and surfaces (patch points) changed the repeatability relative to a fixed landmark-only dataset. We did so for both 3D and µCT datasets to see if these trends differed by scan device.

Finally, we investigated whether there is a difference between devices in a common task: the correct classification of sexual dimorphism (Fig. 2D). We began with a Procrustes ANOVA in R on the symmetric component for the subset of individuals with sex information (*n* = 11 distinct individuals; *n* = 66 3D models). This allowed us to gauge the magnitude of the effect of sexual dimorphism compared to other sources of variation, including variation due to scan device. Then with *Morpho*, we averaged the shape of each replicate triad for each device, and performed a between-group PCA using sex as group (Boulesteix, 2005). Between-group principal component analysis is an ordination technique which is gaining popularity in geometric morphometrics (eg. Firmat et al., 2012; Franchini et al., 2016; Franchini et al., 2014; Fruciano et al., 2016; Fruciano et al., 2014; Mitteroecker & Bookstein, 2011; Raffini, Fruciano & Meyer, 2018; Schmieder et al., 2015; Seetah, Cardini & Miracle, 2012) However, it can be also thought of as a classification tool, as in the *Morpho* implementation which allows performing leave-one-out cross-validation. We, then used cross-validated classification accuracy as a measure of performance in classifying individuals based on their sex.

## Results

### Analyses of shape variation

Our Procrustes ANOVA results indicate that variation among individuals (%Var = 48.3) contributes the most to total variance, with asymmetry (directional and fluctuating), device, and operator error contributing the remainder (Table 1A). The %Var values indicate that directional asymmetry contributes a similar amount of variation as other sources of non-biological variation and that fluctuating asymmetry accounts for much less than digitization error and variation between devices (Table 1A). This means that using analyses of asymmetry with a combination of µCT and 3D surface scans would likely be unreliable in specimens the size of delicate mice or for specimens scanned at a similarly low resolution. The Procrustes ANOVA results for just the 3D data, confirms this observation in which digitization error is large compared to the components of asymmetric variation (Table 1B). For the 3D dataset, the error term (Res/Rep) contributes 17.8% of variation while asymmetry (Side) contributes 19.2%. In other words, for our 3D scan dataset, error contributes almost as much variation as asymmetry (Table 1B). The Procrustes ANOVA for just the µCT dataset, however, did not have this problem to the same degree. Here, the error term (Res/Rep) contributes only 8.52% of variation while asymmetry (Side) contributes 20.8% (Table 1C). In other words, error contributes less than one half of the contribution of asymmetry in the µCT dataset.

**Table 1:** General Procrustes ANOVA on sources of shape variation including asymmetry. The %Var column of this Procrustes ANOVA demonstrates the relative contribution of each factor to overall variation. It is calculated from the sum of squares for each factor divided by the total sum of squares for all factors.

(A) All specimens						
	*Df*	SS	MS	%Var	*F*	Pr(>F)
Individual	8,010	6.21E−02	7.76E−06	48.3	11.2	<.0001
Side	415	2.37E−02	5.70E−05	18.4	82.4	<.0001
Ind * Side	7,470	5.17E−03	6.93E−07	4.02	0.54	1
Device	16,340	2.08E−02	1.27E−06	16.1	4.90	<.0001
Res / Rep	65,360	1.70E−02	2.59E−07	13.2		

(B) Only 3D specimens						
	*Df*	SS	MS	%Var	*F*	Pr(>F)
Individual	8,010	3.52E−02	4.40E−06	51.6	4.24	<.0001
Side	415	1.31E−02	3.15E−05	19.2	30.4	<.0001
Ind * Side	7,470	7.75E−03	1.04E−06	11.4	2.79	<.0001
Res / Rep	32,680	1.22E−02	3.72E−07	17.8		

(C) Only CT specimens						
	*Df*	SS	MS	%Var	*F*	Pr(>F)
Individual	8,010	3.45E−02	4.31E−06	61.7	6.41	<.0001
Side	415	1.17E−02	2.81E−05	20.8	41.8	<.0001
Ind * Side	7,470	5.02E−03	6.72E−07	8.97	4.61	<.0001
Res / Rep	32,680	4.76E−03	1.46E−07	8.52		

The Procrustes ANOVA on just the symmetric component of shape reports the individual shape variation, representing biological variation, is 73.4% (Table 2). Differences between scan devices represent 14.6% and the residuals encompassing differences among replicates or operator error represent 12.0% of total variance (Table 2). Thus, our Procrustes ANOVA on the symmetric component shows that most of the variation is due to biological sources but the significance of the variation due to device may indicate systematic error.

**Table 2:** Procrustes ANOVA on the sources of shape variation using the symmetric component of shape. The R-squared column of this Procrustes ANOVA demonstrates the relative contribution of each factor to overall variation. The shape variation of this dataset is visualized in Figs. 4 and 5.

	*Df*	SS	MS	Rsq	*F*	*Z*	Pr(>F)
ind	18	6.23E−02	3.46E−03	0.734	25.8	21.4	0.001
ind:Dev	19	1.24E−02	6.52E−04	0.146	4.86	23.7	0.001
Residuals	76	1.02E−02	1.34E−04	0.120			
Total	113	8.49E−02					

**Figure 4:**
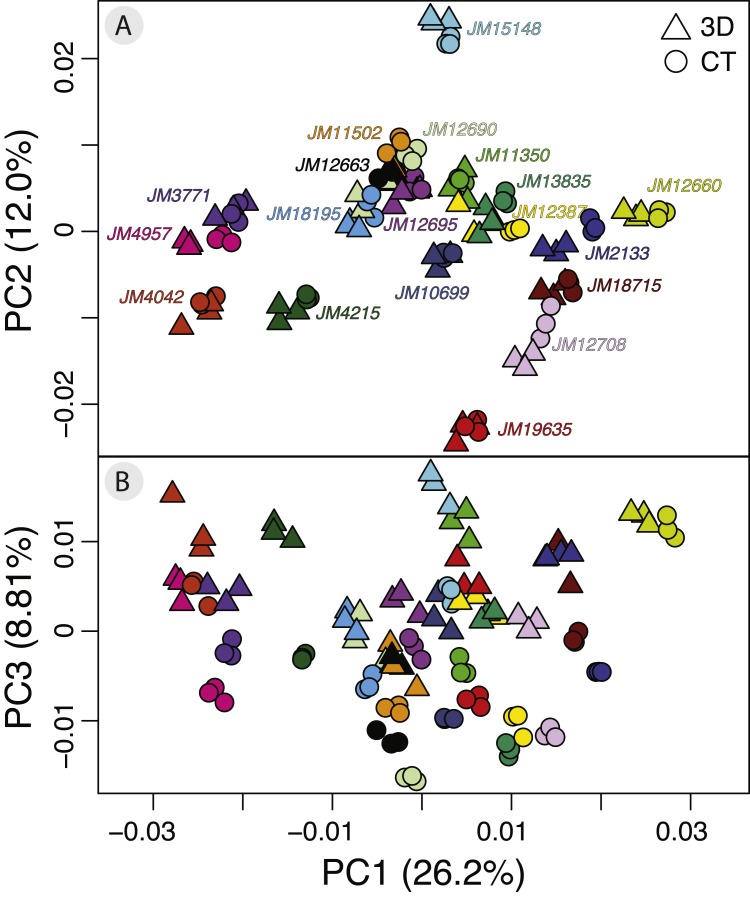
Exploratory PCA plots of shape variation showing differences among individuals, scan devices, and replicates of the same scan device. (A) PC1 versus PC2 and (B) PC1 versus PC3. Each individual has a unique color shared by all of its six replicates. Each individual has three triangles to represent the 3D scanned replicates and 3 circles to represent the µCT scanned replicates. Each axis reports the total variance explained by that principal component.

**Figure 5:**
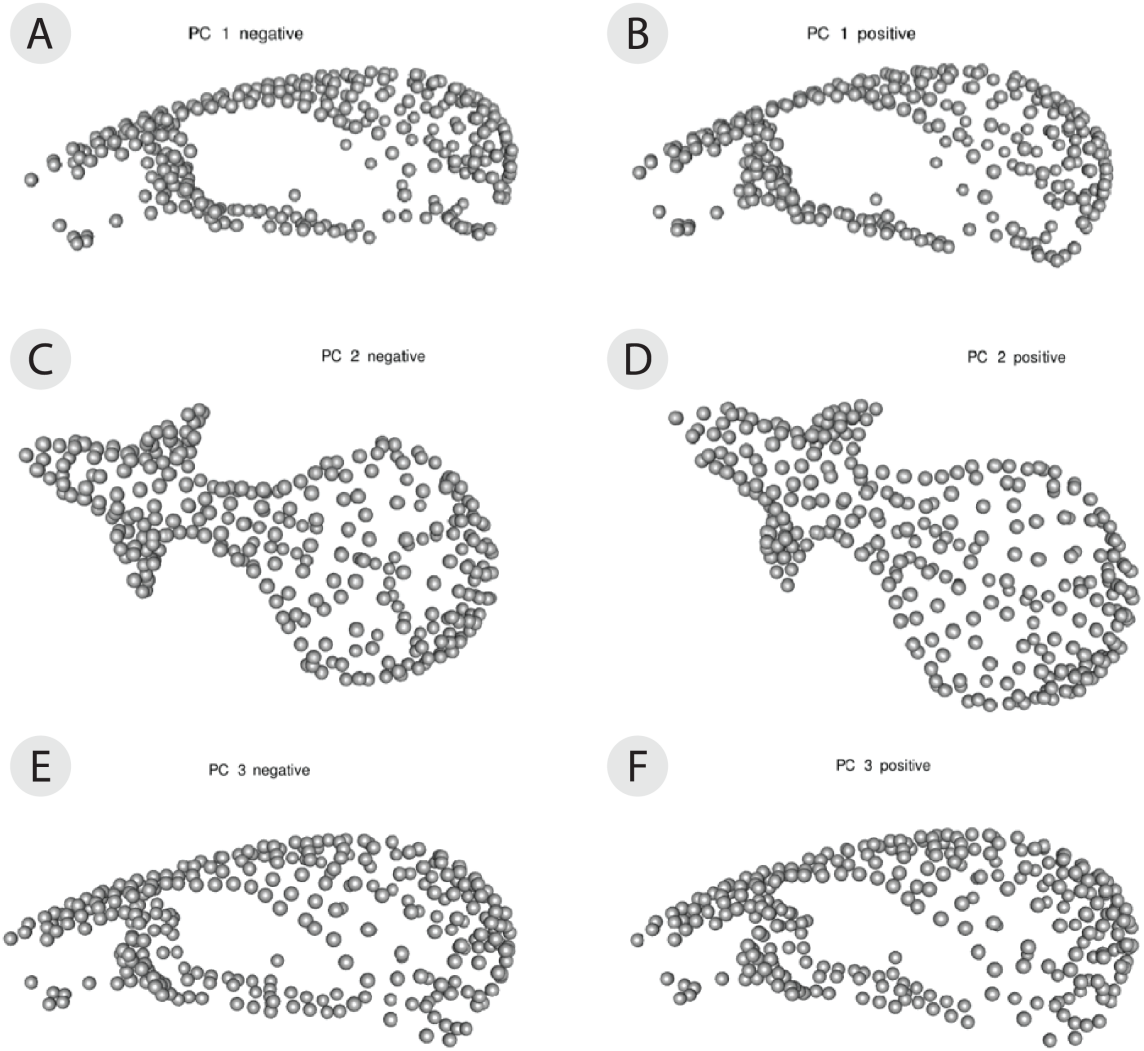
3D warp-grids for the three most important principal components, showing minimum and maximum shapes for each PC. The craniums in (A, C, and E) show the shape of the minimum negative value for each principle component (PC) and the craniums in (B, D, and F) show the shape of the maximum positive value for each PC. Compared to the minimum negative shape (A), more positive values along PC1 (26.4% variance) correspond to a larger braincase relative to the rostrum (B). Compared to the minimum negative shape (C), more positive values along PC2 (11.9% variance) correspond to a wider frontal bone (D). Compared to the minimum negative shape (E), more positive values along PC3 (8.9% variance) correspond to a more dorsally-curved ventral surface (F).

The PCA on the symmetric component revealed that the first three principal components (PCs) account for 47.0% of total variation (PC1 = 26.4%, PC2 = 12.0%, PC3 = 8.81%, *n* = 114) (Fig. 4). Each of the remaining PCs accounted for 6% or less of total variation therefore we only considered the first three for the exploration of patterns of variation. Positive values along PC1 correspond to a larger braincase relative to the rostrum (Fig. 5A). Positive values along PC2 correspond to a wider frontal bone (Fig. 5B). Finally, positive values along PC3 correspond to a more convex, dorsally-curved ventral surface (Fig. 5C).

The plot of the scores on PC1 and PC2 supports the results from the Procrustes ANOVA on the symmetric component of shape in that most of the visible variation is between individuals, i.e., clusters of each individual’s replicates (Fig. 4A). Indeed, regardless of scanning device, replicates from the same individual cluster together (Fig. 4A). For most individuals, replicates occupy non-overlapping regions of the plot except for those around the crowded mean shape near the origin (Fig. 4A). Within each individual’s variation on PCA scores, µCT replicates usually form a tighter cluster than the 3D replicates (Fig. 4A). This pattern suggests that using µCT scans introduces less random error than using 3D scans. Furthermore, within an individual, 3D scan replicates tend to cluster closer to other 3D replicates while µCT scan replicates tend to cluster closer to other µCT replicates (Fig. 4A). Indeed, for most individuals, 3D scan replicates score lower than the µCT scan replicates from the same individual on both PC1 and PC2. These results suggest the systematic error may be driven by µCT scans overestimating both braincase volume and frontal bone width relative to 3D scans (Figs. 4A, 5A, 5B).

Overall, the scores along the first two PCs complement and provide an intuitive visualization for the patterns of higher error in 3D scans and of systematic error between the scan devices as observed in the Procrustes ANOVAs (Tables 1 and 2). The scores along PC1 and PC3 highlight another possible systematic difference between 3D and µCT scans (Fig. 4B). The PC3 axis displaces µCT replicates from 3D replicates such that variation in PC3 scores within individuals is often larger than variation in PC3 scores among individuals (Fig. 4B). On the PC3 axis, almost all 3D scan replicates had higher scores, which correspond to a more dorsally curved ventral surface relative to their corresponding µCT scan replicates (Figs. 4B, 5C).

### Procrustes variance and repeatability

**Figure 6:**
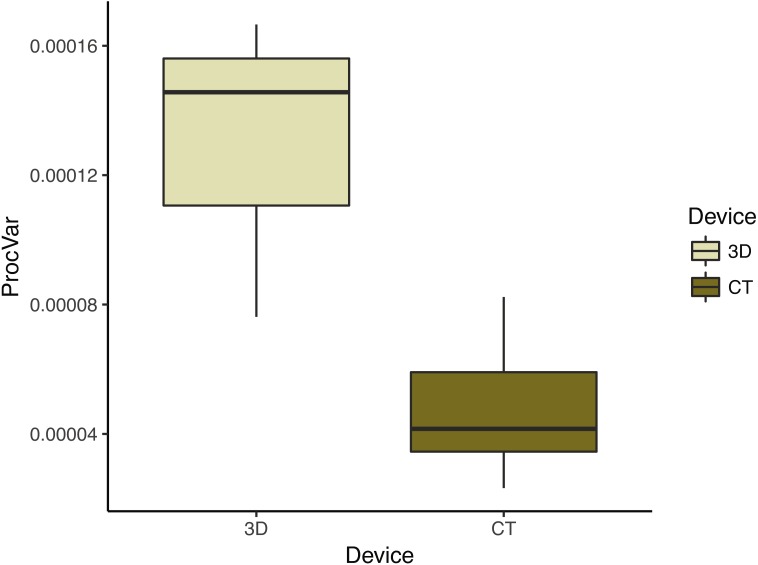
Morphological disparity—as measured by shape variation among replicate scan triads—by scanning device reflects operator error. This box plot summarizes the morphological disparity (also known as the Procrustes variance) among the three replicates of an individual for each scan type. The mean Procrustes variance for 3D scans was 1.34 × 10^−4^ and 4.81 × 10^−5^ for µCT scans. This is a significant difference (*p* < 0.001).

To compare the digitization error in each scanning device dataset, we calculated the Procrustes variance among the replicate triads of each individual. We found that Procrustes variance is significantly (*p* < 0.001) higher in 3D scans (mean = 1.31 ×10^−4^) than in µCT scans (mean = 4.76 ×10^−5^) (Fig. 6). This means that digitizations are more variable in 3D scans than in µCT which is consistent with decreased clustering in 3D scans relative to µCT scans in the PCAs (Fig. 4).

The repeatability for each scan dataset mirrored the Procrustes variance results. We found that the µCT scan dataset had a repeatability of 0.896 and the 3D scan data had a repeatability of 0.750 (Table 3A, Table 3D). This means operators are more successful at repeating their digitizations (i.e., landmark placements) with µCT scans than with 3D scans.

**Table 3:** Comparison of operator error in 3D scan and µCT scan datasets using Procrustes ANOVAs and repeatability scores. The repeatability score (R) is a value that reflects the ease of digitizing in a repeated measure study design. It is calculated from the Procrustes ANOVA using formulas for the intra-class correlation coefficient. The Procrustes ANOVAs were found by subsetting the dataset by scan device and by landmark types and then performing separate generalized Procrustes and bilateral symmetry alignments. (A–C) Results from the 3D-only dataset. (D–F) Results from the µCT-only dataset. (A) and (D) show the repeatabilites from the entire landmark datasets of each scan device. (B) and (E) remove patch points. (C) and (F) contain only fixed landmarks.

(A) 3D scan all landmarks including patches (*n* = 289)								
	*Df*	SS	MS	Rsq	*F*	*Z*	Pr(>F)	*R*
Ind	18	3.53E−02	1.96E−03	0.826	10.0	16.0	0.001	0.750
Residuals	38	7.46E−03	1.96E−04	0.174				
Total	56	4.28E−02						

(B) 3D scan fixed landmarks and semilandmarks (*n* = 203)								
	*Df*	SS	MS	Rsq	*F*	*Z*	Pr(>F)	*R*
Ind	18	4.37E−02	2.43E−03	0.807	8.826	16.7	0.001	0.723
Residuals	38	1.04E−02	2.75E−04	0.193				
Total	56	5.41E−02						

(C) 3D scan fixed landmarks only (*n* = 58)								
	*Df*	SS	MS	Rsq	*F*	*Z*	Pr(>F)	*R*
Ind	18	6.90E−02	3.83E−03	0.749	6.30	16.6	0.001	0.639
Residuals	38	2.31E−02	6.09E−04	0.251				
Total	56	9.21E−02						

(D) CT scan all landmarks including patches (*n* = 289)								
	*Df*	SS	MS	Rsq	*F*	*Z*	Pr(>F)	*R*
Ind	18	3.46E−02	1.92E−03	0.927	26.9	18.4	0.001	0.896
Residuals	38	2.72E−03	7.15E−05	0.073				
Total	56	3.73E−02						

(E) CT scan fixed landmarks and semilandmarks (*n* = 203)								
	*Df*	SS	MS	Rsq	*F*	*Z*	Pr(>F)	*R*
Ind	18	4.33E−02	2.41E−03	0.921	24.7	19.0	0.001	0.888
Residuals	38	3.71E−03	9.76E−05	0.079				
Total	56	4.70E−02						

(F) CT scan fixed landmarks only (*n* = 58)								
	*Df*	SS	MS	Rsq	*F*	*Z*	Pr(>F)	*R*
Ind	18	6.28E−02	3.49E−03	0.893	17.6	20.2	0.001	0.847
Residuals	38	7.54E−03	1.98E−04	0.107				
Total	56	7.03E−02						

To test how different types of landmarks impacted repeatability, we calculated repeatability for combinations of landmark types for 3D and µCT datasets consisting of only the symmetric component of shape (Table 3). Because sliding landmarks depend on the placement of fixed landmarks (and patch points depend on both fixed and semi-landlandmark curves), we could not isolate each type of landmark’s repeatability. The analyses restricted to completely manually placed fixed landmarks always had the lowest repeatability of the three types of landmarks (Table 3C, Table 3F). Repeatability was always highest for the datasets including all three types of landmarks including the semi-automated semi-landmarks and the completely automated patch points (Table 3A, Table 3D). Higher repeatability in datasets with the sliding landmarks may result because the sliding smooths out user placement error across replicates.

### Analyses with a biological example: sexual dimorphism

**Figure 7:**
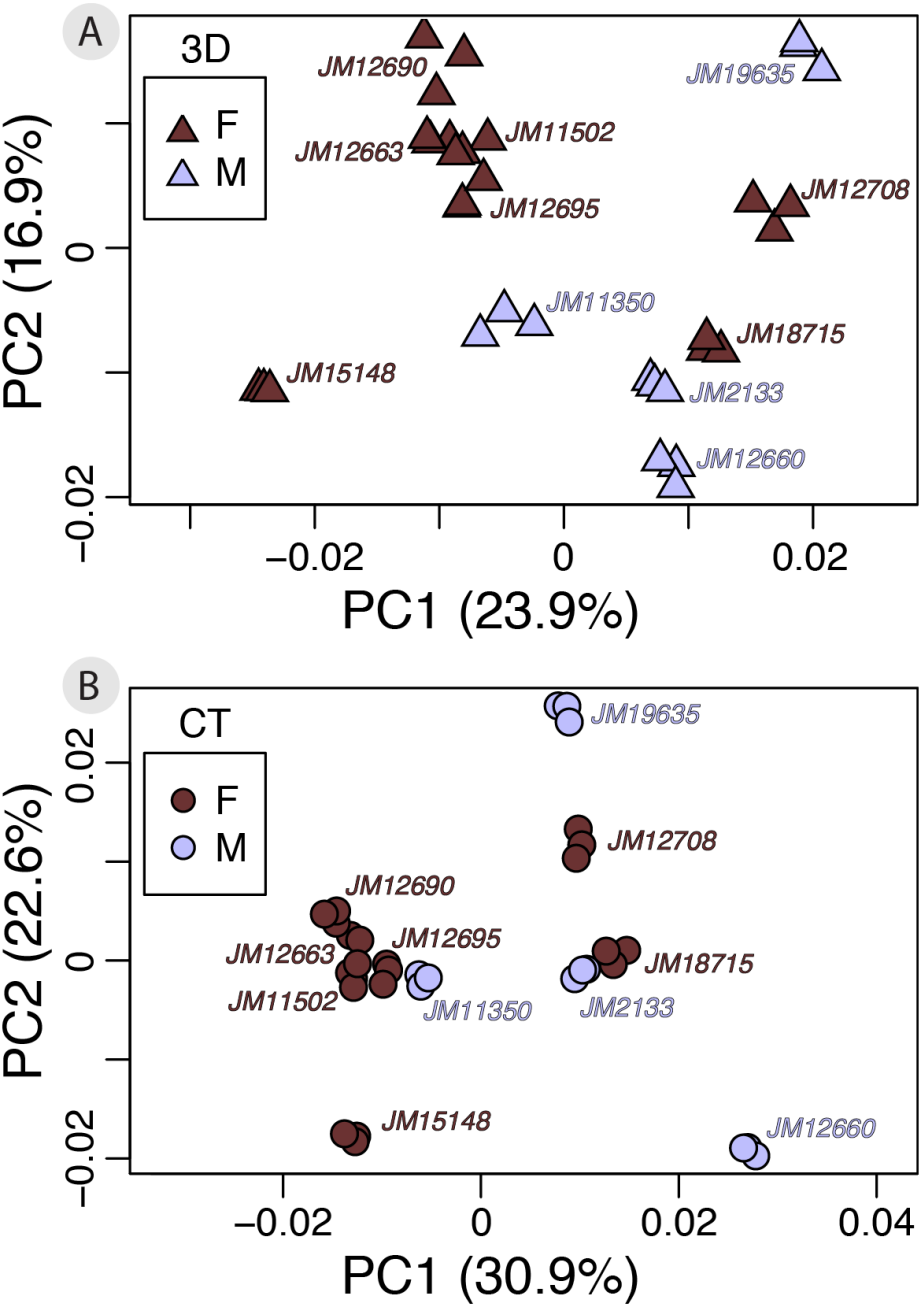
ntra-specific variation as shown by PCAs of 3D (A) and *μ*CT (B) scan datasets colored by sex. Each PCA provides an exploratory visualization of shape variation between males and females in our subsample with sex information (*n* = 11). Males (*n* = 4) are plotted in light blue and females (*n* = 7) are plotted in dark red. Results from the cross-validation test can be found in Table 5.

A small subset of our dataset had sex information (*n* = 11; *f* = 7, *m* = 4), allowing us to perform a test of whether using different scan devices classify males and females according to shape with the same level of accuracy. Our Procrustes ANOVA on the symmetric component of shape variation using sex and device as factors found that shape differences due to device (Rsq = 0.0646) and sex (Rsq = 0.0952) are both significant (*p* < 0.001). Both factors have relatively small effect sizes, however, sex captures slightly more shape variation than device (Table 4). However, the between-group PCAs do not suggest marked sexual dimorphism to begin with (Fig. 7). Therefore, the subtlety of this biological signal could be the main reason for the small contribution of sex to total variation. Finally, we performed a cross-validation test on the between-group PCAs to assess which scan dataset can more reliably classify sexes based on shape (Table 5). The results show that in this case, 3D scans and µCT scans perform identically (overall classification accuracy = 63.6%).

**Table 4:** Symmetric Procrustes ANOVA with device and sex as factors to assess relative contribution of intra-specific variation to overall shape variation. This Procrustes ANOVA allows comparison of the relative contribution to total variation from scan device and sex (R-squared column).

	*Df*	SS	MS	Rsq	*F*	*Z*	Pr(>F)
Device	1	2.99E−03	2.99E−03	0.0646	4.84	4.06	0.001
Sex	1	4.40E−03	4.40E−03	0.0952	7.14	4.96	0.001
Residuals	63	3.88E−02	6.16E−04				
Total	65	4.62E−02					

**Table 5:** Between group PCA classification test to assess whether one scan device dataset performs better at identifying sexes based on shape. This analysis averages shape among replicates, computes a between-group PCA separately for 3D and µCT datasets, and runs a cross-validation classification test. The results indicate whether one type of scan dataset is more successful at classifying males versus females based on the shape variation present in the dataset. It also returns a kappa statistic; a kappa value over 0.20 indicates “fair” agreement between the two datasets. Shape variation visualized by sex can be seen in Fig. 7.

**Cross-validated classification results in frequencies**		
**3D**	**f**	**m**
**f** (***n*** = **7**)	5	2
**m** (***n*** = **4**)	2	2
**CT**	**f**	**m**
**f** (***n*** = **7**)	5	2
**m** (***n*** = **4**)	2	2
**Cross-validated classification results in %**		
**3D**	**f**	**m**
**f**	71.4	28.6
**m**	50.0	50.0
**CT**	**f**	**m**
**f**	71.4	28.6
**m**	50.0	50.0
**Overall classification accuracy (%)**		
**3D**	63.6	
**CT**	63.6	
**Kappa statistic**		
**3D**	0.214	
**CT**	0.214	

## Discussion

In this study, we contrasted very high resolution µCT scans with their extreme opposite: 3D surface scans of very small specimens. Our low versus high resolution datasets allowed us to assess whether the low resolution scans still allow defensible investigations of biological shape variation. We found that despite the low quality of the 3D scans, sufficient amounts of biological variation are present to perform, at the very least, typical interspecific comparisons. In datasets with only very slight intra-specific differences, more difficulties in distinguishing biological signal from the noise introduced by error during data collection. For example, the subtle sexual dimorphism in our small sample was only just distinguished. However, we present three considerations to make before using low resolution datasets. First, we found that variation due to scan device and digitizations is substantial relative to asymmetric variation. This makes low resolution datasets a poor choice for studies on asymmetry. Second, using 3D scans creates more random error due to increased landmarking difficulty, therefore care should be taken in landmark choice, and possibly landmarking software and operator choice. Digitization error may also be reduced by taking averages of repeated measurements (Arnqvist & Martensson, 1998; Fruciano, 2016). Third, using 3D scans also introduces a source of systematic error relative to µCT scans, therefore we recommend not combining them whenever possible (see also Fruciano et al., 2017), and especially in studies on small intra-specific variation. In summary, with a few precautions listed above, we expect that for studies with similarly sized skulls or similarly low resolution scans, the variation due to error will be sufficiently low for successful detection of interspecific shape differences.

### Measurement error and 3D scan reliability

Systematic error between the two scan devices is shown by consistent displacement patterns in the PCA. Indeed, across all three PC axes, the scans differ in how they measure concavity around the braincase, frontal, and ventral surface. This systematic pattern could suggest that the 3D scanner technology errs by adding volume to the digital specimen relative to the µCT scan but it could also be the other way around with the µCT scan distorting the images to reduce volume. Furthermore, even when using the symmetric component of shape, the percent of variation contributed by scan device is quite substantial at about 14.5%. Because scan device contributes this much to variation and because systematic error between scan device exists, researchers expecting very small variation due to biological sources would be advised not to combine 3D scan and µCT scan datasets.

While the two scan devices are usually comparable, using the low resolution 3D scans introduces more digitization error than the higher resolution µCT scans, which likely reflects increased user error due to lower resolution in 3D scans. This increased random error is reflected in both the larger point clouds of 3D replicates relative to µCT replicates in the PCAs, the higher Procrustes variance, and the lower repeatability score of 3D scans, particularly of manually-placed fixed landmarks. As expected, we found that the low resolution 3D scans were more difficult to landmark because key cranial features such as sutures and smaller processes were less distinct (Fig. 1). Nevertheless, our overall 3D scan repeatability score of 0.75 with symmetric data appears consistent with the literature: it is much lower than 3D scanned human-sized skulls—above 0.95 (Badawi-Fayad & Cabanis, 2007; Fourie et al., 2011) but it is approaching the range of 3D scanned macropodoids (e.g., kangaroos)—0.78–0.98, depending on device and landmark choice (Fruciano et al., 2017). This trend of decreasing repeatability with decreasing body size may reflect measurement error becoming a larger percentage of overall size (Robinson & Terhune, 2017). Relatedly, recent work has shown that excluding a few unreliable landmarks, or those with greater variability in placement, can significantly increase repeatability (Fruciano et al., 2017). This may be especially true for small specimens, for which small variations from the landmark location represent a larger percentage of their overall size.

Our repeatability tests on different combinations of landmark types suggest that fixed landmarks suffer the most from decreased resolution and the associated increased user error while patch points suffer the least. We interpret these results to mean that the (semi-) automatic placement of semi-landmark curves and patches is more consistent in placing points compared to a human operator placing fixed landmarks, regardless of whether the automatic placement is “correct” or not. It is important to note that while semi-landmarks were “semi-automated”, the user still manually defined the curve they slid along for each specimen. Furthermore, this curve is bounded by user-placed fixed landmarks. Therefore, the increased repeatability with increasing automation could also be due to the increased degrees of freedom afforded to landmarks during sliding: fixed with zero degrees, semi-landmarks with one degree, and patch points with two. The sliding, by removing variation tangential to a certain direction, will reduce the variance in those points which will appear to vary less so it would be expected that these points will contribute less overall variation when combined with the fixed landmarks.

This study did not look at multiple operator error which can be considerable, particularly if difficult landmarks are included (Fruciano et al., 2017). If inter-operator error were combined with the resolution-driven measurement error found here, it is possible that biological signal would diminish to a degree that could not support even interspecific comparisons.

### Measurement error introduced by scanning device compared to biological variation

The challenge of any quantitative measurement study is to minimize measurement error introduced from various sources (in our case, device, resolution, and observer) relative to the “true” signal of biological variation. For subtle sources of biological variation, such as asymmetry, our results show that the error associated with collecting data from 3D scans contributes the same amount of variation as asymmetry. Therefore, a low resolution study of asymmetry with 3D scans would likely be unreliable unless appropriate arrangements were made to reduce error (Fruciano, 2016), whereas µCT scans may be more suitable for these types of studies. In the case of inter-observer error, which is another common source of measurement error, several studies suggest that interspecific variation can overwhelm inter-observer error such that this does not pose an issue with the correct interpretation of results (Robinson & Terhune, 2017).

In our test on the ability of different scan devices to classify according to sexual dimorphism, we showed that while variation contributed by each source was similar (and that from scan device slightly lower), both scan datasets presented a small sexually dimorphic pattern and supported the same classification performance. This suggests that 3D scans may even be acceptable for detecting some intra-specific patterns. However, this was a small sample (*n* = 11) and further studies with larger datasets would improve confidence for using 3D scans for intra-specific studies. Studies based on larger datasets might also be able to better highlight differences in classification performance between devices, if any. Nevertheless, it is promising that 3D scans and µCT scans performed equally even at such a small sample size for such a subtle intra-specific signal.

### Choosing a digitization device: 3D surface scanning versus *μ*CT versus photogrammetry

With many options for digitizing 3D specimens available, decisions on the acquisition mode must consider price, scanning time, processing time, portability, and scan resolution. The one-off investment of a relatively high resolution 3D surface scanner such as the HDI109 provided a model portable enough to take on airplanes and with fast scanning and processing times. Our model took 10 min from starting the scan to the finished surface file, but note that larger specimens requiring multiple sub-scans will take longer. These fast acquisition times are an asset in collection efforts that rely on expensive and time-limited museum travel. For example, one of us (AEM) digitized over 100 individuals in one week using the same scanning protocol. However, the quality and speed of scanning varies by model; for example, other 3D surface scanners could take over 45 min to capture one specimen and may also require more effort to process scans (Katz & Friess, 2014).

Compared to 3D surface scanners, µCT scanners provide much higher resolution, which in this study translated into less measurement error. However, uCT facilities are not widely accessible, not mobile, and tend to be more expensive. Depending on the facility, µCT scanning involves transport to the facility, scanning either by the operator, processing scans into image stacks, and finally loading scans into specialized (and frequently high-cost) software to do the 3D reconstruction. These reconstructions can be time consuming especially if the cranium needs to be separated from the mandibles. Finally, specimens need to be loaned from their collections for µCT acquisition, which requires specimen transport and curator permission and is particularly difficult when large numbers of specimens from distant locations need to be scanned.

This study did not investigate photogrammetry, which is another and increasingly popular method for digitizing 3D shape. This method uses software to align 2D photographs taken from many different views into a 3D file. Photogrammetry is much cheaper and more portable than 3D surface scanning since it only requires a camera of suitable resolution and very affordable photo-alignment software like Agisoft PhotoScan (Agisoft LLC, St. Petersburg, Russia; http://www.agisoft.com). The trade-offs are that in our experience, photogrammetry takes at least three times longer to acquire the photos, it involves higher risk of human error or inconsistency during photography, and it requires an order of magnitude more time to align the photos into a 3D digital file. While photo-alignment can be done at convenience after photography, the greater time required to capture enough photos may be a deciding factor for researchers with time limitations in museum collections. As for scan resolution, photogrammetry may perform better than 3D surface scanners in some cases (Fourie et al., 2011) or at least provide an acceptable alternative (Katz & Friess, 2014; Muñoz Muñoz, Quinto-Sánchez & González-José, 2016).

Scan resolution is not the only consideration when choosing a scan device as its unique requirements for 3D model processing may increase image noise and therefore landmarking difficulty. Compared to µCT scanning, 3D scans tend to be both noisier and require more model processing before 3D model export. Specifically, artificial smoothing and hole-filling may change the topography of the 3D mesh. Therefore, the comparison we have presented here is not just a comparison of resolutions but also a comparison of 3D model generation. The methods we provide in Supplemental Information 1 represent the settings we found to decrease noise, however, the software also required some model smoothing and hole-filling before export. We recommend that researchers take these additional sources of image modification into account during their landmark choice and study design.

## Conclusions

Here, we have shown that a 3D surface scanner can provide an acceptable alternative to a µCT scanner for assessing biological signal of 3D shape even in small specimens that are at the limits of 3D scanner resolution. Our analyses specifically showed that first, error contributes to a higher percentage of variation in 3D scan datasets than in µCT scan datasets of the same small specimens. As a result, we conclude that 3D scans are usually not appropriate for studies on very small sources of variation like fluctuating asymmetry. Second, we show that 3D scan datasets have a lower repeatability of landmark placement, especially for fixed landmarks, as compared to µCT scans. Relatedly, our comparisons of repeatability on data with asymmetry to the same data without asymmetry—i.e., having bilateral symmetry—support analyzing the bilaterally symmetrical data of landmarks from low resolution scans. Finally, we use a preliminary study of sexual dimorphism to suggest that despite elevated error and shape variance, bilaterally symmetrical datasets from 3D scans can support male versus female classification based on small biological differences as well as µCT datasets can. In summary, while 3D scans are a promising alternative, exploratory pilot studies of measurement error like this one are advisable when practically possible (see also Fruciano, 2016).

Furthermore, the best 3D capture method will vary based on the study’s design, expected effect size for the biological variation of interest, and the researcher’s limitations on time, money, and travel. In addition to image resolution requirements, it is wise to assess the time it will take to capture and process each specimen as well as portability needs. We recommend a preliminary test on multiple devices–including surface scanners–to determine how levels of error compare to biological signal and whether there is substantial systematic error. Doing so may provide a defensible alternative to an expensive and time consuming large-scale acquisition of µCT scans including for studies on very small specimens.

##  Supplemental Information

10.7717/peerj.5032/supp-1 Table S1 Catalogue numbers and sex information for all specimens Catalogue numbers are searchable in the Queensland Museum database, which also provided the sex information for the 19 delicate mouse (*Pseudomys delicatulus*) specimens we used in our study.

10.7717/peerj.5032/supp-2 Table S2 Landmark, semi-landmark curve, and patch point names and definitions There are a total of 289 points used to capture crania shape. 58 are fixed landmarks (LM): the first 12 are centrally located and the remaining 46 come in right and left pairs (right is odd, left is even in the table numbering system). 145 points were placed along 39 curves as semi-landmarks: the first 7 curves are centrally located and the remaining 32 curves come in right and left pairs (right is even, left is odd). 86 points were placed on 12 surface patches, all of which come in right and left pairs (right is even, left is odd).

10.7717/peerj.5032/supp-3 Supplemental Information 1 Supplementary methods for optimal use of an HD109 3D surface scanner for small biological specimens This standard operating procedures outlines the best practices for 3D scanning, which we chose after extensive trial and error.

10.7717/peerj.5032/supp-4 Supplemental Information 2 R script for analyses presented in this study This file should be copied into R, Rstudio, or similar. All datasets required to run the analyses are contained in the supplementary information. Some analyses must be run in MorphoJ, an outside software freely available at: http://www.flywings.org.uk/morphoj_page.htm.

10.7717/peerj.5032/supp-5 Supplemental Information 3 Raw landmark coordinates produced by digitizing 3D mesh files in Viewbox This is the raw format of our shape data produced by exporting the landmark coordinates once landmarking in Viewbox was finished.

10.7717/peerj.5032/supp-6 Supplemental Information 4 Sex identification for specimens in intra-specific analyses This table is nearly identical to Table S1 except that the “Catalogue Number” column heading is shortened to “CatNum” to ease the merging datasets. This dataset is necessary to perform the intra-specific analyses presented here.

10.7717/peerj.5032/supp-7 Supplemental Information 5 Definitions of semilandmarks as required for sliding in geomorph during Procrustes superimposition This table simply encodes the two neighboring points that a semi-landmark can slide between. Point numbers can be related to their position on the cranium using Fig. 3 or Table S2. This table is necessary to treat sliding semi-landmarks correcting during Procrustes alignment using geomorph’s gpagen() function.

10.7717/peerj.5032/supp-8 Supplemental Information 6 Table of all digitized points with bilateral symmetry for symmetric shape analysis This table only includes the points which have bilateral symmetry (i.e., a symmetric pair with one point on the right side of the skull and the other point in the corresponding symmetric location on the left side of the skull). The numbers in the “Right” column are landmarks with their respective bilateral landmark pair in the “Left” column of numbers. Points include landmarks, semi-landmarks, and patch points. Points placed along the skull’s center line are not included as they do not come in symmetric pairs. This table is necessary to isolate the symmetric component of shape in geomorph using the bilat.symmetry() function. See R script for further information and use.

10.7717/peerj.5032/supp-9 Supplemental Information 7 Table of digitized points with bilateral symmetry for symmetric shape analysis for datasets of only fixed landmarks and semi-landmark curve points This table only includes the fixed landmarks and semi-landmark curve points which have bilateral symmetry (i.e., a symmetric pair with one point on the right side of the skull and the other point in the corresponding symmetric location on the left side of the skull). The numbers in the “Right” column are landmarks with their respective bilateral landmark pair in the “Left” column of numbers. Points only include fixed landmarks and semi-landmarks and should only be used with a shape dataset subsetted to exclude patch points. Points placed along the skull’s center line are not included as they do not come in symmetric pairs. This table is necessary to isolate the symmetric component of shape in geomorph using the bilat.symmetry() function. See R script for further information and use.

10.7717/peerj.5032/supp-10 Supplemental Information 8 Table of digitized points with bilateral symmetry for symmetric shape analysis of fixed landmark-only datasets This table only includes the points which are fixed landmarks and have bilateral symmetry (i.e., a symmetric pair with one point on the right side of the skull and the other point in the corresponding symmetric location on the left side of the skull). The numbers in the “Right” column are landmarks with their respective bilateral landmark pair in the “Left” column of numbers. Points only include fixed landmarks and should only be used with a shape dataset subsetted to include only fixed landmarks. Points placed along the skull’s center line are not included as they do not come in symmetric pairs. This table is necessary to isolate the symmetric component of shape in geomorph using the bilat.symmetry() function. See R script for further information and use.

## Abbreviations

LM: Landmark
µCT: Micro-computed tomography
PCA: Principal component analysis
PC: Principal component
3D: Three-dimensional

## Additional Information and Declarations

### Data Availability

The following information was supplied regarding data availability: Morphosource: https://www.morphosource.org/Detail/ProjectDetail/Show/project_id/458.
