# Supplementary material for: Low resolution scans can provide a sufficiently accurate, cost- and time-effective alternative to high resolution scans for 3D shape analyses
Source: PeerJ. 2018 Jun 22;6:e5032. doi: 10.7717/peerj.5032 (PMC6016532; doi:10.7717/peerj.5032)
Supplement: Supplemental Information 1 — This standard operating procedures outlines the best practices for 3D scanning, which we chose after extensive trial and error. [file peerj-06-5032-s003.docx]

**Standard Operating Procedure for Scanning Small Biological Objects with the HD109 Advance Scanner**

**1) Set up**

First, the scanner needs to be plugged in/turned on before the FlexScan3D software can be launched.

Let the scanner equilibrate to working temperature by allowing it to run for 1 hour before starting scans.

**2) Scanning**

Tick the box next to “Rotary” and calibrate the rotary table using the framed grid of black and white squares provided with the scanner.

Place the specimen on the center crosshair in the camera’s field of view — this will be projected onto the surface of the rotary table.

Use the following settings:

- - Generate: Mesh
  - Alignment: None
  - Clean-up: Standard
  - Mesh Density: 99
  - Rotary: 8 (45 degree rotation per scan)
  - Auto Combine: Yes
  - HDR: No
  - High Sensitivity: Yes
  - Exposure: ~60 ms (about 5% overexposed - i.e. the live view shows 5% of specimen in red)

Click SCAN.

Create a cut plane if the camera picks up the rotary table’s screws as this will prevent good alignment later. See page 68 in the HDI109 manual on how to set a cut plane. For us, selecting just 1 screw produced a better result than selecting all four.

Repeat scan settings for each view of the specimen. Refer to the Methods section in the main article text for information about the 3 views we took.

**3) Combining multiple scans**

See page 75 in the FlexScan manual for instruction on how to align scans from different views in the program.

Align scans one after another — i.e. not in pair groups.

When all are aligned, use shift click to select them all and then click Combine.

**4) Exporting the 3D file**

Select the combined file and then click Finalize; use the following settings:

- - Precise Merge
  - Hole Filling: 100
  - Generate: None
  - Decimate: No

Select the dropdown button under export to choose “.stl” (or any other option) as the file type.

Click the finalized file and then click Export to create the .stl file.
