# Supplementary material for: Low resolution scans can provide a sufficiently accurate, cost- and time-effective alternative to high resolution scans for 3D shape analyses
Source: PeerJ. 2018 Jun 22;6:e5032. doi: 10.7717/peerj.5032 (PMC6016532; doi:10.7717/peerj.5032)
Supplement: Supplemental Information 2 — This file should be copied into R, Rstudio, or similar. All datasets required to run the analyses are contained in the supplementary information. Some analyses must be run in MorphoJ, an outside software freely available at: http://www.flywings.org.uk/morphoj_page.htm. [file peerj-06-5032-s004.rtf]

### 3 Oct 2017: Created to analyze methodology study with data from Viewbox### 11 Jan 2018: Cleaned up### 8-16 Feb 2018: Incorporated comments & additions by Dr. Carmelo Fruciano & Dr. Vera Weisbecker to perform analyses on the symmetric component of shape only (including the Procrustes ANOVAs with nested terms) as well as export certain datasets into MorphoJ### 27 Feb 2018: Improved readability of script for peer review### 10-30 Apr 2018: Revised to address reviewer comments and to make greater use of user created functions for better readability##############################################################"########################  Data Set up  ######################"################################################################ Load necessary librarieslibrary(dplyr)library(stringr)library(stringi)library(geomorph)library(Morpho)library(MASS)library(ggplot2)## Read in data file and get it into format that geomorph recognizesmy_data <- read.csv("Raw_coordinates.csv", header = T)# Remove problematic fixed landmarks (these were identified during landmarking as unreliable and removed before any analyses were run)temp <- my_data[-(907:912),] # A pt of ext auditory meatustemp <- temp[-(889:894),] # A pt squamosal / Alisphenoidtemp <- temp[-(877:882),] # AL premax&maxillar intersecttemp <- temp[-(847:852),] # Bullae&basioccciotal/basisphenoidtemp <- temp[-(769:774),] # Deepest pt aud bullaetemp <- temp[-(652:657),] # L pt of occipital condyletemp <- temp[-(508:513),] # Lat pt foramen under infraorbitalmy_data <- temp[-(283:288),] # P pt of foramen ovale# This leaves 58 landmarks, 145 semilandmarks, and 86 patches for a total of 289 pointsdata <- my_data[, 2:(dim(my_data)[2])] # gets rid of LM names for geomorph formatting reasonsdata <- t(data)A <- arrayspecs(data, 289, 3) # convert to 3D array with 289 points and 3 dimensions## Create function to scrape filenames for metadatametadata <- function(threeDarray, cols) {        names <- dimnames(threeDarray)[[3]]        names <- gsub("ind", "", names)        categories <- strsplit(names, "_")         my_classifiers <- matrix(unlist(categories), ncol = length(cols), byrow = T)         colnames(my_classifiers) <- cols        sp_info <- as.data.frame(my_classifiers)        sp_info$Both <- with(sp_info, interaction(CatNum, Type, sep = "_"))        sp_info$All <- with(sp_info, interaction(CatNum, Type, Rep, sep = "_"))        return(sp_info)}# Create metadata tablecols = c("CatNum", "Type", "Rep") # Define default column namessp_info <- metadata(A, cols)## Remove problem specimen JM14056# The meshes for this specimen were mirror-imaged before landmarking making the 3D and CT scans not comparablegood_data <- (sp_info$CatNum != "JM14056")A <- A[ , , good_data]sp_info <- sp_info[good_data, ]sp_info$Order <- c(1:nrow(sp_info)) # preserve landmarking order## Create vectors of patch points, semi-landmarks, and landmarks# LM point name metadatapt_names <-my_data[, 1] # grabs first column of raw coordsfoo2 <- str_sub(pt_names, 3, -1) # gets rid of X, Y, Z designationsu_pt_names <- unique(foo2)patches <- str_detect(u_pt_names, "PAT")pat_num <- which(patches == TRUE)sliders <- str_detect(u_pt_names, "DCUR")sli_num <- which(sliders == TRUE)LM <- !(sliders | patches)LM_num <- which(LM == TRUE)## Load in bilateral landmarks and sliders# For all landmarks land.pairs <- read.csv("Bilateral_Landmarks.csv", header = T)sli_matrix <- read.csv("Smatrix.csv", header = T)# For semi-landmarks and fixed landmarks onlyLM_SM_pairs <- read.csv("Bilateral_LM_SM_only.csv", header = T)# Convert all-point slider matrix into one without patchesLM_SM_matrix <- sli_matrixfor (k in 1:dim(sli_matrix)[2]){        for (i in 1:dim(sli_matrix)[1]){               original <- u_pt_names[sli_matrix[i,k]]               LM_SM_matrix[i,k] <- which(u_pt_names_LM_SM == original)        }}# For fixed landmarks onlyLM_only_pairs <- read.csv("Bilateral_LM_only.csv", header = T)##############################################################"################### Prepare Datasets  #######################"################################################################# Datasets with asymmetry## Perform procrustes alignment in R with geomorph for all dataY <- gpagen(A, Proj = TRUE, ProcD = TRUE, curves = sli_matrix, surfaces = pat_num)## 3D only subset is_td <- (sp_info$Type == "3D")td_set <- A[, ,is_td]td_sp_info <- sp_info[is_td, ]td_Y <- gpagen(td_set, Proj = TRUE, ProcD = TRUE, curves = sli_matrix, surfaces = pat_num)## CT only subset is_ct <- (sp_info$Type == "CT")ct_set <- A[, ,is_ct]ct_sp_info <- sp_info[is_ct, ]ct_Y <- gpagen(ct_set, Proj = TRUE, ProcD = TRUE, curves = sli_matrix, surfaces = pat_num)### Datasets with bilateral symmetry## All databY <- bilat.symmetry(Y$coords, ind = sp_info$All, object.sym = T, replicate = NULL, side = NULL, land.pairs = land.pairs)ind.coords <- bY$symm.shape # Analogous to Y$coords# Create new table of specimen information since the bilat function rearranges the coordinate arrayb_names <- metadata(ind.coords, cols)## 3D only bilateral subsettd_bY <- bilat.symmetry(td_Y, ind = td_sp_info$All, object.sym = T, replicate = NULL, side = NULL, land.pairs = land.pairs)td_ind.coords <- td_bY$symm.shapetd_b_names <- metadata(td_ind.coords, cols)## CT only bilateral subsetct_bY <- bilat.symmetry(ct_Y, ind = ct_sp_info$All, object.sym = T, replicate = NULL, side = NULL, land.pairs = land.pairs)ct_ind.coords <- ct_bY$symm.shapect_b_names <- metadata(ct_bY$symm.shape, cols)### Sex-only Datasetspse_sex <- read.csv("Pse_sex.csv", header = T)sex <- dplyr::filter(pse_sex, Sex != "u")sp_info_sex <- merge(sp_info, sex, by = "CatNum", all.x = FALSE)good_set <- A[,,sp_info_sex$Order] # subsets the coordinate data AND re-orders it so they match# Redo gpa & bilateral symmetry with sexed-only datasetY_sex <- gpagen(good_set, Proj = TRUE, ProcD = TRUE, curves = sli_matrix, surfaces = pat_num)bY_sex <- bilat.symmetry(Y_sex, ind = sp_info_sex$All, object.sym = T, replicate = NULL, side = NULL, land.pairs = land.pairs)ind.coords_sex <- bY_sex$symm.shape# Create new table of specimen information since the bilat function rearranges the coordinate arrayb_names_sex <- metadata(ind.coords_sex, cols)b_names_sex$CatNum <- str_replace(b_names_sex$CatNum, "ind", "")b_names_sex$Order <- c(1:nrow(b_names_sex))b_names_sex <- merge(b_names_sex, sex, by = "CatNum", all.x = F)b_names_sex$Class <- with(b_names_sex, interaction(All, Sex, sep = "_"))ind.coords_sex <- ind.coords_sex[,,b_names_sex$Order]dimnames(ind.coords_sex)[[3]] <- b_names_sex$Class## Subset sexed-only dataset into scan device datasets# 3D scansbcoordsSex_3D <- ind.coords_sex[,,which(b_names_sex$Type=="3D")]b_nameSex_3D <- b_names_sex[which(b_names_sex$Type=="3D"),]# CT scansbcoordsSex_CT <- ind.coords_sex[,,which(b_names_sex$Type == "CT")]b_nameSex_CT <- b_names_sex[which(b_names_sex$Type == "CT"),]##############################################################"########################  Analyses  #########################"##############################################################"### 1) MorphoJ Procrustes ANOVAs ###""####################################"### Table 1 ##### Import all data into MorphoJ, where error is designed as a nested factor in ANOVAsr2morphoj(Y$coords,"DataForTable1.txt")## Analyze complete dataset in MorphoJ:# Make a new project with "DataForTable1.txt", tick 3D and object symmetry# Perform: Preliminaries > New Procrustes Fit with alignment by principal axis# If any pairings are inconsistent use the Bilateral_Landmark.csv doc to fix# Perform: Preliminaries > Extract New Classifier from ID string, do so for CatNum (1, 7), Device (-4, -3), and Rep (-1, -1)# Perform: Variation > Procrustes ANOVA with CatNum as Individual, Device as Error 1 and Rep as Error 2# Scroll up to see results reported in Table 1## Analyze each device type separately# 3D onlyr2morphoj(td_Y$coords,"DataForTable1b.txt")# Follow instructions for "Analyze in MorphoJ" above using new table except put "Rep" as Error 1 and leave "Device" out# CT onlyr2morphoj(ct_Y$coords,"DataForTable1c.txt")# Follow instructions for "Analyze in MorphoJ" above using new table except put "Rep" as Error 1 and leave "Device" out"### 2) Symmetric Procrustes ANOVA ###""#####################################"### Table 2 ##### Run Procrustes ANOVA on bilaterally symmetric dataDFforGeneralProcANOVA <- geomorph.data.frame(coords = ind.coords, ind = factor(b_names$CatNum), Dev = b_names$Type) GeneralProcANOVA <- procD.lm(coords ~ ind + Dev %in% ind, data = DFforGeneralProcANOVA, iter = 999)GeneralProcANOVA # Results reported in Table 2write.csv(GeneralProcANOVA$aov.table, "Table2.csv")# Calculate variation explained among repetitions within scan typeGeneralProcANOVA$SS[3]/GeneralProcANOVA$SS[4]"### 3) Symmetric Exploratory PCA ###""####################################"### Figures 4 & 5 and Table TK ##### Graph the PCA with the symmetric component of shape# Assign colorsgp <- as.factor(b_names$CatNum)col.gp <- c("#001AFFFF", "#00FF19FF", "#FF9900FF", "#FFE500FF", "#CCFF00FF", "black", "#33FF00FF", "#FF4D00FF", "#00FF66FF", "#00FFB2FF", "#00FFFFFF", "#00B3FFFF", "dark red", "#FF0000FF", "#3300FFFF","#7F00FFFF", "#CC00FFFF", "dark green", "#FF0099FF")names(col.gp) <- levels(gp)col.gp <- col.gp[match(gp, names(col.gp))] # colors by CatNum# Assign point types by device, 3D as triangles, CT as circlesdev.gp <- rep("21", length(col.gp))dev.gp[which(b_names$Type == "3D")] <- "24"dev.gp <- as.numeric(dev.gp)## Plot PCAspca <- plotTangentSpace(ind.coords, groups = col.gp, axis1 = 1, axis2 = 2, label = NULL, verbose = T) # change axis1 to see warpplots for different PCs## Look at variation explained by each PCpca# Check for outliersoutliers <- plotOutliers(ind.coords)# Look at outliersis <- which(b_names$Both == "JM12660_CT")plotRefToTarget(ind.coords[,,5], ind.coords[,,24], method = "vector", label = F)b_names[5,]# PC1 vs PC2plot(pca$pc.scores[,1], pca$pc.scores[,2], asp = T, xlab = "PC1 (26.2%)", ylab = "PC2 (12.0%)", pch = dev.gp, cex = 1.5, bg = col.gp, cex.axis = 1.3, cex.lab = 1.3)axis(side = 1, at = c(-0.2,0.1), labels = FALSE, col.ticks = "white")legend(0.025, 0.025, legend = c("CT", "3D"), col = "black", pch = c(21, 24))text(pca$pc.scores[,1], pca$pc.scores[,2], labels = b_names$CatNum, cex = 0.7)# PC1 vs PC3plot(pca$pc.scores[,1], pca$pc.scores[,3], asp=T, xlab= "PC1 (26.2%)", ylab= "PC3 (8.81%)", pch = dev.gp, cex = 1.5, bg = col.gp, cex.axis = 1.3, cex.lab = 1.3)axis(side = 1, at = c(-0.2,0.1), labels = FALSE, col.ticks = "white")#text(pca$pc.scores[,1], pca$pc.scores[,3], labels = b_names$CatNum, cex = 0.7)## Both graphs were cleaned up for better readability in Illustrator"### 4) Repeatability ###""########################"### Table 3 ##### Define function for repeatability using formulas 1-3 in Fruciano 2016# Function takes in a 3D array of shape coordinates, a list of identifiers, the number of repetitions taken for each individual, and has options to print the ANOVA table and export this table along with the repeatability value to a csv called "filename"rep_ability <- function(coords, ind, nRep, print = TRUE, export = FALSE, filename = NULL) {        r.gdf <- geomorph.data.frame(coords = coords, ind = factor(ind))        rep.er <- procD.lm(coords ~ ind, data = r.gdf, iter = 999)                S_sq_A <- ((rep.er$aov.table$MS[1] - rep.er$aov.table$MS[2]) / nRep) # Among-individuals variance component        S_sq_W <- rep.er$aov.table$MS[2] # Within-ind variance component        R <- S_sq_A / (S_sq_W + S_sq_A) # Analogue of the intraclass correlation coeffiecent (i.e. uses multivariate data) also known as repeatability in the GMM field                table <- rep.er$aov.table        table$Repeatability <- R        if (print) {                print(rep.er$aov.table)                cat("\n","Repeatability =", R)        }        if (export) {                write.csv(table, file = paste(filename, ".csv", sep = ""))        }}## Calculate repeatability for individual+scan type on dataset with bilateral symmetry# Repeatability for 3D scans onlyrep_ability(td_ind.coords, td_b_names$CatNum, nRep = 3, print = T, export = T, "Rep_ability_bilat_3D")# Repeatability for CT scans onlyrep_ability(ct_ind.coords, ct_b_names$CatNum, 3, T, T, "Rep_ability_bilat_CT")"### 5) Repeatability by landmark type ###""#########################################"## Define function that subsets the shape coordinate array by LM type, redoes GPA and bilateral symmetry, and returns repeatability; requires a 3D array of landmark coordinates, the numbers for the landmarks included, an optional slider matrix, the appropriate bilateral landmark table, and a filename label for the output table.RepByLMtype <- function(Array, info, nRep, LMnums, slider_matrix, LM_pairs, Label) {        LM_set <- Array[LMnums, ,]        Y <- gpagen(LM_set, Proj = TRUE, ProcD = TRUE, curves = slider_matrix)                bY_LM <- bilat.symmetry(Y$coords, ind = info$All, object.sym = TRUE, replicate = NULL, side = NULL, land.pairs = LM_pairs)        LM_ind.coords <- bY_LM$symm.shape                names <- metadata(LM_ind.coords, cols)                rep_ability(LM_ind.coords, names$CatNum, nRep, T, T, Label)}## Run repeatabilities by device type and landmark type# 3D datasetsRepByLMtype(td_set, td_sp_info, 3, c(LM_num, sli_num), LM_SM_matrix, LM_SM_pairs, "3D Fixed and Semi LMs")RepByLMtype(td_set, td_sp_info, 3, LM_num, NULL, LM_only_pairs, "3D Fixed LMs Only")# CT datasetsRepByLMtype(ct_set, ct_sp_info, 3, c(LM_num, sli_num), LM_SM_matrix, LM_SM_pairs, "CT Fixed and Semi LMs")RepByLMtype(ct_set, ct_sp_info, 3, LM_num, NULL, LM_only_pairs, "CT Fixed LMs Only")"### 6) Analysis of Disparity ###""################################"### Figure 6 and in-text reports of p-value and mean variance ##### Make individual/type combos a factor to use the model's residuals to assess intra-observer error using the dataset with only the symmetric shape componentCat <- as.factor(b_names$Both)Dev <- as.factor(b_names$Type)gdf_CF <- geomorph.data.frame(ind.coords, Cat = Cat, Dev = Dev)Disparity <- morphol.disparity(ind.coords ~ Cat, groups = NULL, iter = 999) ## Create box plot of disparity that shows how variances within each Device/Individual combination compare - i.e. how operator error within replicates comparesDisparityFactor <- as.factor(unlist(lapply(names(Disparity$Procrustes.var), function(x) stri_sub(x,-2))))ProcVarCF <- data.frame(ProcVar = Disparity$Procrustes.var, Device = DisparityFactor)BoxPlot <- ggplot(ProcVarCF, aes(x = Device, y = ProcVar, fill = Device)) +         geom_boxplot() +        theme_classic()BoxPlot # Results shown in Figure 6dev.off()## Test for differences in variance and get P-values for those differencesDisparity2 <- morphol.disparity(ind.coords ~ Cat, groups = ~Dev, iter = 999)Disparity2 # Results reported in text"### 7) Procrustes ANOVA w Sex ###""#################################"### Table 4 ##### Run Procrustes ANOVA on sex as a separate factor from device and residualsgdf <- geomorph.data.frame(sex = b_names_sex$Sex, device = b_names_sex$Type)procD_Sex <- procD.lm(ind.coords_sex ~ device + sex, data = gdf)print(procD_Sex)write.csv(procD_Sex$aov.table, "ProcD_Sex_and_Device.csv")"### 8) Cross Validation Classification Tests ###""################################################"### Table 5 ##### Calculate the mean coordinates for each specimen for CT dataMeanCoords_CTsex <- aggregate(vecx(bcoordsSex_CT), by = list(b_nameSex_CT$CatNum), FUN = mean) # Update metadata to reflect just one of each specimen and take only the CatNum and Sex columns mb_nameSex_CT <- unique(b_nameSex_CT[,c(1,7)])rownames(mb_nameSex_CT) <- mb_nameSex_CT$CatNumrownames(MeanCoords_CTsex) <- MeanCoords_CTsex$Group.1mb_nameSex_CT <- mb_nameSex_CT[rownames(MeanCoords_CTsex),]## Calculate the mean coordinates for each specimen for 3D dataMeanCoords_3Dsex <- aggregate(vecx(bcoordsSex_3D), by = list(b_nameSex_3D$CatNum), FUN = mean) # Update metadata to reflect just one of each specimen and take only the CatNum and Sex columnsmb_nameSex_3D <- unique(b_nameSex_3D[,c(1,7)])rownames(mb_nameSex_3D) <- mb_nameSex_3D$CatNumrownames(MeanCoords_3Dsex) <- MeanCoords_3Dsex$Group.1mb_nameSex_3D <- mb_nameSex_3D[rownames(MeanCoords_3Dsex),]## Create the between group PCAs_bwgPCA_CT <- groupPCA(MeanCoords_CTsex[,2:ncol(MeanCoords_CTsex)], mb_nameSex_CT$Sex, rounds = 10000, cv = T, weighting = F)s_bwgPCA_3D <- groupPCA(MeanCoords_3Dsex[,2:ncol(MeanCoords_3Dsex)], mb_nameSex_3D$Sex, rounds = 10000, cv = T, weighting = F)# Results reported in Table 5s_bwgPCA_3Ds_bwgPCA_CT"### 9) bwgPCAs of Sex Data ###""##############################"### Figure 7 ### ## Make PCA of sexed-only 3D datagp <- as.factor(b_nameSex_3D$Sex)col.gp <- c("red","blue", "grey") # Color points by sex (f, m, unknown)names(col.gp) <- levels(gp)col.gp <- col.gp[match(gp, names(col.gp))] pca <- plotTangentSpace(bcoordsSex_3D, groups = col.gp, pch = 24, axis1 = 1, axis2 = 2, label = NULL, verbose = T)# Fancy plotplot(pca$pc.scores[,1], pca$pc.scores[,2], asp = T, xlab = "PC1 (23.9%)", ylab = "PC2 (16.9%)", pch = 24, cex = 1.5, bg = col.gp, cex.axis = 1.3, cex.lab = 1.3)axis(side = 1, at = c(-0.2,0.1), labels = FALSE, col.ticks = "white")legend(-0.035, 0.015, legend = c("F", "M"), col = c("red", "blue"), pch = 24)text(pca$pc.scores[,1], pca$pc.scores[,2], labels = b_nameSex_3D$CatNum, cex = 0.7)## Repeat for PCA of sexed-only  CT datagp <- as.factor(b_nameSex_CT$Sex)col.gp <- c("red","blue", "grey")names(col.gp) <- levels(gp)col.gp <- col.gp[match(gp, names(col.gp))] pca <- plotTangentSpace(bcoordsSex_CT, groups = col.gp, axis1 = 1, axis2 = 2, label = NULL, verbose = T)# Fancy plotplot(pca$pc.scores[,1], pca$pc.scores[,2], asp = T, xlab = "PC1 (30.9%)", ylab = "PC2 (22.6%)", pch = 21, cex = 1.5, bg = col.gp, cex.axis = 1.3, cex.lab = 1.3)axis(side = 1, at = c(-0.2,0.1), labels = FALSE, col.ticks = "white")legend(-0.035, 0.015, legend = c("F", "M"), col = c("red", "blue"), pch = 21)text(pca$pc.scores[,1], pca$pc.scores[,2], labels = b_nameSex_CT$CatNum, cex = 0.7)# Additional aesthetic changes were made in Illustrator
